# Generalized Distance-Based Entropy and Dimension Root Entropy for Simplified Neutrosophic Sets

**DOI:** 10.3390/e20110844

**Published:** 2018-11-04

**Authors:** Wen-Hua Cui, Jun Ye

**Affiliations:** Department of Electrical engineering and Automation, Shaoxing University, 508 Huancheng West Road, Shaoxing 312000, China

**Keywords:** simplified neutrosophic set, simplified neutrosophic generalized distance-based entropy, simplified neutrosophic dimension root entropy, decision making

## Abstract

In order to quantify the fuzziness in the simplified neutrosophic setting, this paper proposes a generalized distance-based entropy measure and a dimension root entropy measure of simplified neutrosophic sets (NSs) (containing interval-valued and single-valued NSs) and verifies their properties. Then, comparison with the existing relative interval-valued NS entropy measures through a numerical example is carried out to demonstrate the feasibility and rationality of the presented generalized distance-based entropy and dimension root entropy measures of simplified NSs. Lastly, a decision-making example is presented to illustrate their applicability, and then the decision results indicate that the presented entropy measures are effective and reasonable. Hence, this study enriches the simplified neutrosophic entropy theory and measure approaches.

## 1. Introduction

Since entropy is an effective measure approach in quantifying the uncertainty degree of the objects, with the development of fuzzy theory, a lot of research on fuzzy entropy has been done so far. Zadeh [1] first defined fuzzy entropy for fuzzy sets regarding the probability distribution of a fuzzy event. Then, De-Luca and Termini [2] formulated axioms of fuzzy entropy and proposed a non-probabilistic logarithm of fuzzy entropy. Exponential fuzzy entropy was presented by Pal and Pal [3]. Yager [4] put forward the metric distance-based entropy by measuring the lack of distinction between the fuzzy set and its complement. The weighted fuzzy entropy with trigonometric functions of membership degree was constructed by Parkash and Sharma [5]. Thereafter, the generalized parametric exponential fuzzy entropy of order-α was introduced by Verma and Sharma [6], which reduces to the Pal and Pal exponential entropy [3] when α = 1, and becomes the De-Luca and Termini logarithmic entropy [2] when α → 0. However, for an intuitionistic fuzzy set (IFS) extended by adding a non-membership degree to a fuzzy set (FS), Burillo and Bustince [7] first proposed IFS and interval-valued IFS entropy measures and their axiom requirements. Then, Szmidt and Kacprzyk [8] redefined De-Luca and Termini’s axioms [2] in IFS setting and presented an intuitionistic non-probabilistic fuzzy entropy measure by a geometric interpretation and a ratio of distance of IFSs. Valchos and Sergiadis [9] constructed a new entropy logarithm of IFS on the basis of the De-Luca and Termini fuzzy entropy logarithm [2]. As an extension of logarithmic entropy [2], Zhang and Jiang [10] proposed vague entropy by the intersection and union of the non-membership degree and membership degree for vague sets, and defined vague cross-entropy for IFSs. Further, the cosine and sine entropy of IFS was defined by Ye [11]. An exponential entropy measure of IFS was proposed by Verma and Sharma [12], and then intuitionistic fuzzy entropy was proposed corresponding to the order-α [13] and R-norm [14]. Additionally, for an interval-valued IFS (IvIFS), Ye [15] put forward the sine and cosine entropy of IvIFS. Wei et al. [16] also presented entropy and similarity measures of IvIFS and described their relationships. Then, Zhang et al. [17] defined the distance-based entropy of IvIFS and its relative axiom requirement. Tian et al. [18] proposed a pair of generalized entropy measure on IFSs and IvIFSs.

Recently, the neutrosophic set (NS) was introduced to describe the uncertainty and inconsistency information by an indeterminacy degree added to the IFS. After that, a single-valued NS (SvNS), an interval-valued NS (IvNS), and a simplified NS containing SvSN and IvNS were proposed as subsets of NS and successively used for practical applications. To measure the fuzziness of the NSs, Majumder and Samanta [19] developed the entropy of SvNSs. Aydoğdu [20] introduced the entropy and similarity measures of IvNSs. Then Ye and Du [21] put forward the distances, entropy, and similarity measures of IvNSs and depicted their relationship. Ye and Cui further proposed exponential entropy [22] and sine entropy [23] for simplified NSs. However, some distances-based entropy measures are not developed for simplified NSs in existing literature. Hence, it is necessary to add some distances-based entropy measures of simplified NSs as their complement.

Motivated by distance measures and dimension root similarity measure [24], we proposed the generalized distance-based entropy and dimension root entropy of simplified NSs in this paper. As for the framework of this paper, we introduce some concepts of simplified NSs in Section 2, and then Section 3 proposes the simplified neutrosophic generalized distance-based entropy and dimension root entropy. In Section 4, the comparative analysis of entropy measures for IvNSs is carried out to show the effectiveness and rationality of the presented entropy measures. In Section 5, a decision-making example is used to illustrate the applicability of the novel entropy measures. Lastly, the conclusions and future work of this study are given in Section 6.

## 2. Simplified Neutrosophic Sets

Simplified NS, which contains both SvNS and IvNS, was presented by Ye [25] as a subset of NS for convenient application. Assume there is a universal set *A* = {*a*_1_, *a*_2_, ..., *a_n_*}, then a simplified NS *B* in *A* can be given by *B* = {<*a_i_*, *T_B_*(*a_i_*), *U_B_*(*a_i_*), *F_B_*(*a_i_*)> | *a_i_* ∊ *A*}, where *B* is a SvNS if *T_B_*(*a_i_*), *U_B_*(*a_i_*), *F_B_*(*a_i_*) ∊ [0, 1] and 0 ≤ *T_B_*(*a_i_*) + *U_B_*(*a_i_*) + *F_B_*(*a_i_*) ≤ 3, whereas *B* is an IvNS if *T_B_*(*a_i_*) = $\left[T_{B}^{-}(a_{i}),T_{B}^{+}(a_{i})\right]$, *U_B_*(*a_i_*) = $\left[U_{B}^{-}(a_{i}),U_{B}^{+}(a_{i})\right]$, *F_B_*(*a_i_*) = $\left[F_{B}^{-}(a_{i}),F_{B}^{+}(a_{i})\right]$ with the conditions of $\left[T_{B}^{-}(a_{i}),T_{B}^{+}(a_{i})\right]$, $\left[U_{B}^{-}(a_{i}),U_{B}^{+}(a_{i})\right]$, $\left[F_{B}^{-}(a_{i}),F_{B}^{+}(a_{i})\right]$ Í [0, 1] and $0\leq T_{B}^{+}(a_{i})+U_{B}^{+}(a_{i})+F_{B}^{+}(a_{i})\leq 3$.

Provided that there are two simplified NSs *B* = {<*a_i_*, *T_B_*(*a_i_*), *U_B_*(*a_i_*), *F_B_*(*a_i_*)> | *a_i_* ∊ *A*} and *C* = {<*a_i_*, *T_C_*(*a_i_*), *U_C_*(*a_i_*), *F_C_*(*a_i_*)> | *a_i_* ∊ *A*}, then some operations between *B* and *C* can be given as follows [25,26]:

(1) The sufficient and necessary condition of *B* ⊆ *C* for SvNSs is *T_B_*(*a_i_*) ≤ *T_C_*(*a_i_*), *U_B_*(*a_i_*) *≥ U_C_*(*a_i_*), and *F_B_*(*a_i_*) *≥ F_C_*(*a_i_*), while that for IvNSs is $T_{B}^{-}(a_{i})\leq T_{C}^{-}(a_{i})$, $T_{B}^{+}(a_{i})\leq T_{C}^{+}(a_{i})$, $U_{B}^{-}(a_{i})\geq U_{C}^{-}(a_{i})$, $U_{B}^{+}(a_{i})\geq U_{C}^{+}(a_{i})$, $F_{B}^{-}(a_{i})\geq F_{C}^{-}(a_{i})$, and $F_{B}^{+}(a_{i})\geq F_{C}^{+}(a_{i})$;
(2) The sufficient and necessary condition of *B* = *C* is *B* ⊆ *C* and *C* ⊆ *B*;
(3) The complement of a SvNS *B* is $B^{c}=\{<a_{i},F_{B}(a_{i}),1-U_{B}(a_{i}),T_{B}(a_{i})>\;|a_{i}\in A\}$, and then that of an IvNS *B* is $B^{c}=\{<a_{i},[F_{B}^{-}(a_{i}),F_{B}^{+}(a_{i})],[1-U_{B}^{+}(a_{i}),1-U_{B}^{-}(a_{i})],[T_{B}^{-}(a_{i}),T_{B}^{+}(a_{i})]>\;|a_{i}\in A\}$;
(4) If *B* and *C* are SvNSs, then:
  $$B\cup C=\left\{<a_{i},T_{B}(a_{i})\vee T_{C}(a_{i}),U_{B}(a_{i})\wedge U_{C}(a_{i}),F_{B}(a_{i})\wedge F_{C}(a_{i})>\;|a_{i}\in A\right\},$$
  $$B\cap C=\left\{<a_{i},T_{B}(a_{i})\wedge T_{C}(a_{i}),U_{B}(a_{i})\vee U_{C}(a_{i}),F_{B}(a_{i})\vee F_{C}(a_{i})>\;|a_{i}\in A\right\},$$
  $$B⊕C=\left\{<a_{i},T_{B}(a_{i})+T_{C}(a_{i})-T_{B}(a_{i})T_{C}(a_{i}),U_{B}(a_{i})U_{C}(a_{i}),F_{B}(a_{i})F_{C}(a_{i})>\;|a_{i}\in A\right\},$$
  $$B⊗C=\left\{\langle a_{i},T_{B}(a_{i})T_{C}(a_{i}),U_{B}(a_{i})+U_{C}(a_{i})-U_{B}(a_{i})U_{C}(a_{i}),F_{B}(a_{i})+F_{C}(a_{i})-F_{B}(a_{i})F_{C}(a_{i})\rangle |a_{i}\in A\right\},$$
  $$\gamma B=\left\{<a_{i},1-{\left(1-T_{B}(a_{i})\right)}^{\gamma },U_{B}^{\gamma }(a_{i}),F_{B}^{\gamma }(a_{i})>\;|a_{i}\in A\right\}\;\mathrm{for}\;\gamma >0,$$
  $$B^{\gamma }=\left\{\langle a_{i},T_{B}^{\gamma }(a_{i}),1-{\left(1-U_{B}(a_{i})\right)}^{\gamma },1-{\left(1-F_{B}(a_{i})\right)}^{\gamma }\rangle |a_{i}\in A\right\}\;\mathrm{for}\;\gamma >0.$$

However, if *B* and *C* are IvNSs, then:

$$B\cup C=\left\{\langle \begin{array}{l} a_{i},[T_{B}^{-}(a_{i})\vee T_{C}^{-}(a_{i}),T_{B}^{+}(a_{i})\vee T_{C}^{+}(a_{i})],\\ {}[U_{B}^{-}(a_{i})\wedge U_{C}^{-}(a_{i}),U_{B}^{+}(a_{i})\wedge U_{C}^{+}(a_{i})],\\ \left[F_{B}^{-}(a_{i})\wedge F_{C}^{-}(a_{i}),F_{B}^{+}(a_{i})\wedge F_{C}^{+}(a_{i})\right] \end{array}\rangle |a_{i}\in A\right\},$$

$$B\cap C=\left\{\langle \begin{array}{l} a_{i},[T_{B}^{-}(a_{i})\wedge T_{C}^{-}(a_{i}),T_{B}^{+}(a_{i})\wedge T_{C}^{+}(a_{i})],\\ {}[U_{B}^{-}(a_{i})\vee U_{C}^{-}(a_{i}),U_{B}^{+}(a_{i})\vee U_{C}^{+}(a_{i})],\\ \left[F_{B}^{-}(a_{i})\vee F_{C}^{-}(a_{i}),F_{B}^{+}(a_{i})\vee F_{C}^{+}(a_{i})\right] \end{array}\rangle |a_{i}\in A\right\},$$

$$B⊕C=\left\{\langle \begin{array}{l} a_{i},[T_{B}^{-}(a_{i})+T_{C}^{-}(a_{i})-T_{B}^{-}(a_{i})T_{C}^{-}(a_{i}),T_{B}^{+}(a_{i})+T_{C}^{+}(a_{i})-T_{B}^{+}(a_{i})T_{C}^{+}(a_{i})],\\ \left[U_{B}^{-}(a_{i})U_{C}^{-}(a_{i}),U_{B}^{+}(a_{i})U_{C}^{+}(a_{i})],[F_{B}^{-}(a_{i})F_{C}^{-}(a_{i}),F_{B}^{+}(a_{i})F_{C}^{+}(a_{i})\right] \end{array}\rangle |a_{i}\in A\right\},$$

$$B⊗C=\left\{\langle \begin{array}{l} a_{i},[T_{B}^{-}(a_{i})T_{C}^{-}(a_{i}),T_{B}^{+}(a_{i})T_{C}^{+}(a_{i})],\\ {}[U_{B}^{-}(a_{i})+U_{C}^{-}(a_{i})-U_{B}^{-}(a_{i})U_{C}^{-}(a_{i}),U_{B}^{+}(a_{i})+U_{C}^{+}(a_{i})-U_{B}^{+}(a_{i})U_{C}^{+}(a_{i})],\\ \left[F_{B}^{-}(a_{i})+F_{C}^{-}(a_{i})-F_{B}^{-}(a_{i})F_{C}^{-}(a_{i}),F_{B}^{+}(a_{i})+F_{C}^{+}(a_{i})-F_{B}^{+}(a_{i})F_{C}^{+}(a_{i})\right] \end{array}\rangle |a_{i}\in A\right\},$$

$$\gamma B=\left\{\langle \begin{array}{l} a_{i},[1-{\left(1-T_{B}^{-}(a_{i})\right)}^{\gamma },1-{\left(1-T_{B}^{+}(a_{i})\right)}^{\gamma }],\\ \left[{\left(U_{B}^{-}(a_{i})\right)}^{\gamma },{\left(U_{B}^{+}(a_{i})\right)}^{\gamma }],[{\left(F_{B}^{-}(a_{i})\right)}^{\gamma },{\left(F_{B}^{+}(a_{i})\right)}^{\gamma }\right] \end{array}\rangle |a_{i}\in A\right\}\;\mathrm{for}\;\gamma >0,$$

$$B^{\gamma }=\left\{\langle \begin{array}{l} a_{i},[{\left(T_{B}^{-}(a_{i})\right)}^{\gamma },{\left(T_{B}^{+}(a_{i})\right)}^{\gamma }],[1-{\left(1-U_{B}^{-}(a_{i})\right)}^{\gamma },1-{\left(1-U_{B}^{+}(a_{i})\right)}^{\gamma }],\\ \left[1-{\left(1-F_{B}^{-}(a_{i})\right)}^{\gamma },1-{\left(1-F_{B}^{+}(a_{i})\right)}^{\gamma }\right] \end{array}\rangle |a_{i}\in A\right\}\;\mathrm{for}\;\gamma >0.$$

## 3. Simplified Neutrosophic Generalized Distance-based Entropy and Dimension Root Entropy

In this section, two novel simplified neutrosophic entropy measures, containing a simplified neutrosophic generalized distance-based entropy measure and a simplified neutrosophic dimension root entropy measure, are defined below.

### 3.1. Simplified Neutrosophic Generalized Distance-Based Entropy

**Definition** **1.** *Assume a simplified NS H in a universal set A = {a_1_, a_2_, ..., a_n_} is H = {<a_i_, T_H_(a_i_), U_H_(a_i_), F_H_(a_i_)> | a_i_ ∊ A}. Then, a new generalized distance-based entropy measure of H can be defined as:*

(1) $$EA_{1}^{\rho }(H)=\frac{1}{3n}\sum_{i=1}^{n}\left[\begin{array}{l} 1-2^{\rho }|T_{H}(a_{i})-0.5{|}^{\rho }+1-2^{\rho }|U_{H}(a_{i})-0.5{|}^{\rho }\\ +1-2^{\rho }|F_{H}(a_{i})-0.5{|}^{\rho } \end{array}\right]\;\mathrm{for}\;\mathrm{the}\;\mathrm{SvNS}\;H\;\mathrm{and}\;\rho >0,$$

(2) $$EA_{2}^{\rho }(H)=\frac{1}{6n}\sum_{i=1}^{n}\left[\begin{array}{l} 1-2^{\rho }|T_{H}^{-}(a_{i})-0.5{|}^{\rho }+1-2^{\rho }|T_{H}^{+}(a_{i})-0.5{|}^{\rho }\\ +1-2^{\rho }|U_{H}^{-}(a_{i})-0.5{|}^{\rho }+1-2^{\rho }|U_{H}^{+}(a_{i})-0.5{|}^{\rho }\\ +1-2^{\rho }|F_{H}^{+}(a_{i})-0.5{|}^{\rho }+1-2^{\rho }|F_{H}^{-}(a_{i})-0.5{|}^{\rho } \end{array}\right]\;\mathrm{for}\;\mathrm{the}\;\mathrm{IvNS}\;H\;\mathrm{and}\;\rho >0,$$

*where ρ is an integer value.*

According to the axiomatic definition of the IvNS entropy measure [21], the proposed generalized distance-based entropy measure of a simplified NS has the theorem below.

**Theorem** **1.** *Set A as a universal set A = {a_1_, a_2_, ..., a_n_}. Assume there is a fuzziest simplified NS B = {b_1_, b_2_, ..., b_n_} = {<a_i_, T_B_(a_i_), U_B_(a_i_), F_B_(a_i_)> | a_i_ ∊ A} in the universal set A along with each element b_i_ = <a_i_, 0.5, 0.5, 0.5> (i = 1, 2, ..., n) for SvNS or b_i_ = <a_i_, [0.5, 0.5], [0.5, 0.5], [0.5, 0.5]> (i = 1, 2, ..., n) for IvNS. Then the entropy measure $EA_{k}^{\rho }(H)$ (k = 1, 2; ρ > 0) of the simplified NS H = {h_1_, h_2_, ..., h_n_} = {<a_i_, T_H_(a_i_), U_H_(a_i_), F_H_(a_i_)> | a_i_ ∊ A} satisfies the following properties:*

*(EAP1) $EA_{k}^{\rho }(H)=0$ (k = 1, 2; ρ > 0) if H is a crisp set whose element is <a_i_, 1, 0, 0> or <a_i_, 0, 0, 1> (i = 1, 2, ..., n) for SvNS and <a_i_, [1, 1], [0, 0], [0, 0]> or <a_i_, [0, 0], [0, 0], [1, 1]> for IvNS;*

*(EAP2) $EA_{k}^{\rho }(H)=1$ (k = 1, 2; ρ > 0) if and only if h_i_ = b_i_ for i = 1, 2, ..., n;*

*(EAP3) If one simplified NS H is closer to the fuzziest simplified NS B than the other simplified NS L, then H is fuzzier than L with $EA_{k}^{\rho }(L)<EA_{k}^{\rho }(H)$ (k = 1, 2; ρ > 0);*

*(EAP4) If the complement of H is H^C^, then $EA_{k}^{\rho }(H)=EA_{k}^{\rho }(H^{c})$ (k = 1, 2; ρ > 0).*

**Proof.**  (EAP1) If a crisp set *H* = {*h*_1_, *h*_2_, ..., *h_n_*} is a SvNS with *h_i_* = <*a_i_*, 1, 0, 0> or *h_i_* = <*a_i_*, 0, 0, 1> (*i* = 1, 2, ..., *n*), by Equation (1) we can obtain:

$$\begin{array}{ll} EA_{1}^{\rho }(H) & =\frac{1}{3n}\sum_{i=1}^{n}\left[1-2^{\rho }|T_{H}\left(a_{i}\right)-0.5{|}^{\rho }+1-2^{\rho }|U_{H}\left(a_{i}\right)-0.5{|}^{\rho }+1-2^{\rho }|F_{H}\left(a_{i}\right)-0.5{|}^{\rho }\right]\\ & =\frac{n}{3n}[1-2^{\rho }|1-0.5{|}^{\rho }+1-2^{\rho }|0-0.5{|}^{\rho }+1-2^{\rho }|0-0.5{|}^{\rho }]=0 \end{array}$$

$$\mathrm{or}\;\begin{array}{ll} EA_{1}^{\rho }(H) & =\frac{1}{3n}\sum_{i=1}^{n}\left[1-2^{\rho }|T_{H}\left(a_{i}\right)-0.5{|}^{\rho }+1-2^{\rho }|U_{H}\left(a_{i}\right)-0.5{|}^{\rho }+1-2^{\rho }|F_{H}\left(a_{i}\right)-0.5{|}^{\rho }\right]\\ & =\frac{n}{3n}[1-2^{\rho }|0-0.5{|}^{\rho }+1-2^{\rho }|0-0.5{|}^{\rho }+1-2^{\rho }|1-0.5{|}^{\rho }]=0 \end{array},\mathrm{for}\;\rho >0$$

while if *H* = {*h*_1_, *h*_2_, ..., *h_n_*} is an IvNS with *h_i_* = <*a_i_*, [1, 1], [0, 0], [0, 0]> or *h_i_* = <*a_i_*, [0, 0], [0, 0], [1, 1]> (*i* = 1, 2, ..., *n*), by Equation (2) we have:

$$\begin{array}{ll} EA_{2}^{\rho }(H) & =\frac{1}{6n}\sum_{i=1}^{n}\left[\begin{array}{l} 1-2^{\rho }|T_{H}^{-}\left(a_{i}\right)-0.5{|}^{\rho }+1-2^{\rho }|T_{H}^{+}\left(a_{i}\right)-0.5{|}^{\rho }\\ +1-2^{\rho }|U_{H}^{-}\left(a_{i}\right)-0.5{|}^{\rho }+1-2^{\rho }|U_{H}^{+}\left(a_{i}\right)-0.5{|}^{\rho }\\ +1-2^{\rho }|F_{H}^{-}\left(a_{i}\right)-0.5{|}^{\rho }+1-2^{\rho }|F_{H}^{+}\left(a_{i}\right)-0.5{|}^{\rho } \end{array}\right]\\ & =\frac{1}{6n}\sum_{i=1}^{n}\left[\begin{array}{l} 1-2^{\rho }|1-0.5{|}^{\rho }+1-2^{\rho }|1-0.5{|}^{\rho }\\ +1-2^{\rho }|0-0.5{|}^{\rho }+1-2^{\rho }|0-0.5{|}^{\rho }\\ +1-2^{\rho }|0-0.5{|}^{\rho }+1-2^{\rho }|0-0.5{|}^{\rho } \end{array}\right]=0 \end{array},$$

or:

$$\begin{array}{ll} EA_{2}^{\rho }(H) & =\frac{1}{6n}\sum_{i=1}^{n}\left[\begin{array}{l} 1-2^{\rho }|T_{H}^{-}\left(a_{i}\right)-0.5{|}^{\rho }+1-2^{\rho }|T_{H}^{+}\left(a_{i}\right)-0.5{|}^{\rho }\\ +1-2^{\rho }|U_{H}^{-}\left(a_{i}\right)-0.5{|}^{\rho }+1-2^{\rho }|U_{H}^{+}\left(a_{i}\right)-0.5{|}^{\rho }\\ +1-2^{\rho }|F_{H}^{-}\left(a_{i}\right)-0.5{|}^{\rho }+1-2^{\rho }|F_{H}^{+}\left(a_{i}\right)-0.5{|}^{\rho } \end{array}\right]\\ & =\frac{1}{6n}\sum_{i=1}^{n}\left[\begin{array}{l} 1-2^{\rho }|0-0.5{|}^{\rho }+1-2^{\rho }|0-0.5{|}^{\rho }\\ +1-2^{\rho }|0-0.5{|}^{\rho }+1-2^{\rho }|0-0.5{|}^{\rho }\\ +1-2^{\rho }|1-0.5{|}^{\rho }+1-2^{\rho }|1-0.5{|}^{\rho } \end{array}\right]=0 \end{array}\;\mathrm{for}\;\rho >0.$$

(EAP2) Let $f(x_{i})=1-2^{\rho }|x_{i}-0.5{|}^{\rho }$ (*ρ* > 0) be a function for *x_i_* ∊ [0, 1] (*i* = 1, 2, ..., *n*). Find the extreme values of *f*(*x_i_*) on the closed interval [0, 1] using calculus technique.

At first, by removing the absolute symbol, the function *f*(*x_i_*) can be expressed as:

$$f(x_{i})=\{\begin{array}{c} \begin{array}{l} 1-2^{\rho }{\left(0.5-x_{i}\right)}^{\rho },for\;0\leq x_{i}<0.5\\ 1,\;for\;x_{i}=0.5 \end{array}\\ 1-2^{\rho }{\left(x_{i}-0.5\right)}^{\rho },\;for\;0.5<x_{i}\leq 1 \end{array}\;\mathrm{for}\;\rho >0.$$

For *ρ* = 1, the first derivative of *f*(*x_i_*) with respect to *x_i_* apart from *x_i_ =* 0.5 is:

(3) $$f’(x_{i})=\frac{df(x_{i})}{dx_{i}}=\{\begin{array}{c} 2,for\;0\leq x_{i}<0.5\\ -2,\;for\;0.5<x_{i}\leq 1 \end{array}\;\mathrm{for}\;\rho =1.$$

It is clear that *f*(*x_i_*) is monotonically increasing when *x_i_* ∊ [0, 0.5) and decreasing when *x_i_* ∊ (0.5, 1]. Thus, for the interval [0, 1], *f*(*x_i_*) *=* 1 get the maximum value at the critical point of *x_i_* = 0.5 for *ρ* = 1.

Then, when *ρ* is not equal to 1, the first derivative of *f*(*x_i_*) with respect to *x_i_* can be calculated by:

(4) $$f’(x_{i})=\frac{df(x_{i})}{dx_{i}}=\{\begin{array}{l} 2^{\rho }\rho {\left(0.5-x_{i}\right)}^{\rho -1},for\;0\leq x_{i}<0.5\\ 0,\;for\;x_{i}=0.5\\ -2^{\rho }\rho {\left(x_{i}-0.5\right)}^{\rho -1},\;for\;0.5<x_{i}\leq 1 \end{array}.$$

Obviously, the first derivative of *f*(*x_i_*) is equal to zero only at the point of *x_i_* = 0.5. Because *f’*(*x_i_*) is positive for 0 ≤ *x_i_* < 0.5 and negative for 0.5 < *x_i_* ≤ 1, the maximum of *f*(*x_i_*) = 1 on the closed interval [0, 1] can be obtained at the critical point *x_i_* = 0.5.

Regarding the definition of *f*(*x_i_*), the entropy measure of simplified NS *H* = {*h*_1_, *h*_2_, ..., *h_n_*} = {<*a_i_*, *T_H_*(*a_i_*), *U_H_*(*a_i_*), *F_H_*(*a_i_*)> | *a_i_* ∊ *A*} can be defined as:

$$EA_{1}^{\rho }(H)=\frac{1}{3n}\sum_{i=1}^{n}\left[f(T_{H}(a_{i}))+f(U_{H}(a_{i}))+f(F_{H}(a_{i})\right)]\;\mathrm{for}\;\mathrm{the}\;\mathrm{SvNS}\;H,$$

$$EA_{2}^{\rho }(H)=\frac{1}{6n}\sum_{i=1}^{n}\left[\begin{array}{l} f(T_{H}^{-}(a_{i}))+f(T_{H}^{+}(a_{i}))\\ +f(U_{H}^{-}(a_{i}))+f(U_{H}^{+}(a_{i}))\\ +f(F_{H}^{-}(a_{i}))+f(F_{H}^{+}(a_{i})) \end{array}\right]\;\mathrm{for}\;\mathrm{the}\;\mathrm{IvNS}\;H.$$

It is clear that if and only if *h_i_* = <*a_i_*, 0.5, 0.5, 0.5>, the maximum value of the entropy measure is $EA_{1}^{\rho }(H)=1$ and if and only if *h_i_* = <*a_i_*, [0.5, 0.5], [0.5, 0.5], [0.5, 0.5]>, the maximum value of the entropy measure is $EA_{2}^{\rho }(H)=1$.

(EAP3) According to Equations (3) and (4), *f*(*x_i_*) is monotonically increasing when *x_i_* ∊ [0, 0.5], and monotonically decreasing when *x_i_* ∊ [0.5, 1]. Therefore, the closer the simplified NS *H* is to the fuzziest set *B* than *L*, the fuzzier *H* is than *L* with $EA_{k}^{\rho }(L)<EA_{k}^{\rho }(H)$ (*k* = 1, 2; *ρ* > 0).

(EAP4) When the complement of the SvNS *H* = {<*a_i_*, *T_H_*(*a_i_*), *U_H_*(*a_i_*), *F_H_*(*a_i_*)> | *a_i_* ∊ *A*} is *H^C^* = {<*a_i_*, *F_H_*(*a_i_*), 1 – *U_H_*(*a_i_*), *T_H_*(*a_i_*)> | *a_i_* ∊ *A*}, by Equation (1) we can obtain:

$$\begin{array}{l} EA_{1}^{\rho }(H^{C})=\frac{1}{3n}\sum_{i=1}^{n}\left[1-2^{\rho }|F_{H}(a_{i})-0.5{|}^{\rho }+1-2^{\rho }|1-U_{H}(a_{i})-0.5{|}^{\rho }+1-2^{\rho }|T_{H}(a_{i})-0.5{|}^{\rho }\right]\\ =\frac{1}{3n}\sum_{i=1}^{n}\left[1-2^{\rho }|T_{H}(a_{i})-0.5{|}^{\rho }+1-2^{\rho }|U_{H}(a_{i})-0.5{|}^{\rho }+1-2^{\rho }|F_{H}(a_{i})-0.5{|}^{\rho }\right]=EA_{1}^{\rho }(H) \end{array},\mathrm{for}\;\rho >0.$$

When the complement of the IvNS $H=\left\{<a_{i},[T_{H}^{-}(a_{i}),T_{H}^{+}(a_{i})],[U_{H}^{-}(a_{i}),U_{H}^{+}(a_{i})],[F_{H}^{-}(a_{i}),F_{H}^{+}(a_{i})]>|a_{i}\in A\right\}$ is $H^{C}=\left\{<a_{i},[F_{H}^{-}(a_{i}),F_{H}^{+}(a_{i})],[1-U_{H}^{-}(a_{i}),1-U_{H}^{+}(a_{i})],[T_{H}^{-}(a_{i}),T_{H}^{+}(a_{i})]>\;|a_{i}\in A\right\}$, we can also have $EA_{2}^{\rho }(H^{C})=EA_{2}^{\rho }(H)$.

Thus, the proof of the Theorem 1 is completed.  □

### 3.2. Simplified Neutrosophic Dimension Root Entropy

For two SvNSs *B* = {<*a_i_*, *T_B_*(*a_i_*), *U_B_*(*a_i_*), *F_B_*(*a_i_*)> | *a_i_* ∊ *A*} and *C* = {<*a_i_*, *T_C_*(*a_i_*), *U_C_*(*a_i_*), *F_C_*(*a_i_*)> | *a_i_* ∊ *A*} in the universal set *A*, Ye [24] defined a dimension root distance of SvNSs as follows:

$$D(B,C)=\frac{1}{n}\sum_{i=1}^{n}{\left[\frac{{\left(T_{B}(a_{i})-T_{C}(a_{i})\right)}^{2}+{\left(U_{B}(a_{i})-U_{C}(a_{i})\right)}^{2}+{\left(F_{B}(a_{i})-F_{C}(a_{i})\right)}^{2}}{3}\right]}^{\frac{1}{3}}.$$

Based on the dimension root distance, we can present simplified neutrosophic dimension root entropy for a simplified NS.

**Definition** **2.** *Assume H = {<a_i_, T_B_(a_i_), U_B_(a_i_), F_B_(a_i_)> | a_i_ ∊ A} is a simplified NS in a universal set A = {a_1_, a_2_, ..., a_n_}. Then, we can define the following dimension root entropy measure for the simplified NS H:*

(5) $$EB_{1}(H)=1-\frac{1}{n}\sum_{i=1}^{n}{\left[\frac{4{\left(T_{H}(a_{i})-0.5\right)}^{2}+4{\left(U_{H}(a_{i})-0.5\right)}^{2}+4{\left(F_{H}(a_{i})-0.5\right)}^{2}}{3}\right]}^{\frac{1}{3}}\mathrm{for}\;\mathrm{the}\;\mathrm{SvNS}\;H,$$

(6) $$EB_{2}(H)=1-\frac{1}{n}\sum_{i=1}^{n}{\left[\frac{\begin{array}{l} 4{\left(T_{H}^{-}(a_{i})-0.5\right)}^{2}+4{\left(T_{H}^{+}(a_{i})-0.5\right)}^{2}\\ +4{\left(U_{H}^{-}(a_{i})-0.5\right)}^{2}+4{\left(U_{H}^{+}(a_{i})-0.5\right)}^{2}\\ +4{\left(F_{H}^{-}(a_{i})-0.5\right)}^{2}+4{\left(F_{H}^{+}(a_{i})-0.5\right)}^{2} \end{array}}{6}\right]}^{\frac{1}{3}}\mathrm{for}\;\mathrm{the}\;\mathrm{IvNS}\;H.$$

Similar to the proposed simplified neutrosophic distance-based entropy, the dimension root entropy of simplified NSs also has the following theorem.

**Theorem** **2.** *Assume there is a fuzziest simplified NS B = {b_1_, b_2_, ..., b_n_} = {<a_i_, T_B_(a_i_), U_B_(a_i_), F_B_(a_i_)> | a_i_ ∊ A} in the universal set A = {a_1_, a_2_, ..., a_n_} with each element b_i_ = <a_i_, 0.5, 0.5, 0.5> (i = 1, 2, ..., n) for SvNS or b_i_ = <a_i_, [0.5, 0.5], [0.5, 0.5], [0.5, 0.5]> (i = 1, 2, ..., n) for IvNS. Then the entropy measure EB_k_(H) (k = 1, 2) of the simplified NS H = {h_1_, h_2_, ..., h_n_} = {<a_i_, T_H_(a_i_), U_H_(a_i_), F_H_(a_i_)> | a_i_ ∊ A} satisfies the following properties:*

*(EBP1) EB_k_(H) = 0 if H = {h_1_, h_2_, ..., h_n_} is a crisp set with each element h_i_ = <a_i_, 1, 0, 0> or h_i_ = <a_i_, 0, 0, 1> (i = 1, 2, ..., n) for SvNS, and h_i_ = <a_i_, [1, 1], [0, 0], [0, 0]> or h_i_ = <a_i_, [0, 0], [0, 0], [1, 1]> (i = 1, 2, ..., n) for IvNS;*

*(EBP2) EB_k_(H) = 1 if and only if h_i_ = b_i_ (i = 1, 2, ..., n);*

*(EBP3) If one simplified NS H is closer to the fuzziest simplified NS B than the other simplified NS L, then H is fuzzier than L with EB_k_(L) < EB_k_(H) for k = 1, 2;*

*(EBP4) EB_k_(H) = EB_k_(H^C^) if H^C^ is the complement of H.*

**Proof.**  (EBP1) For a crisp SvNS *H* = {*h*_1_, *h*_2_, ..., *h_n_*} with *h_i_* = <*a_i_,* 1, 0, 0> or *h_i_* = <*a_i_,* 0, 0, 1> (*i* = 1, 2, ..., *n*), by Equation (5) we can obtain:

$$\begin{array}{ll} EB_{1}(H) & =1-\frac{1}{n}\sum_{i=1}^{n}{\left[\frac{4{\left(T_{H}(a_{i})-0.5\right)}^{2}+4{\left(U_{H}(a_{i})-0.5\right)}^{2}+4{\left(F_{H}(a_{i})-0.5\right)}^{2}}{3}\right]}^{\frac{1}{3}}\\ & =1-\frac{1}{n}\sum_{i=1}^{n}{\left[\frac{4{\left(1-0.5\right)}^{2}+4{\left(0-0.5\right)}^{2}+4{\left(0-0.5\right)}^{2}}{3}\right]}^{\frac{1}{3}}=0 \end{array}$$

$$\mathrm{or}\;\begin{array}{ll} EB_{1}(H) & =1-\frac{1}{n}\sum_{i=1}^{n}{\left[\frac{4{\left(T_{H}(a_{i})-0.5\right)}^{2}+4{\left(U_{H}(a_{i})-0.5\right)}^{2}+4{\left(F_{H}(a_{i})-0.5\right)}^{2}}{3}\right]}^{\frac{1}{3}}\\ & =1-\frac{1}{n}\sum_{i=1}^{n}{\left[\frac{4{\left(0-0.5\right)}^{2}+4{\left(0-0.5\right)}^{2}+4{\left(1-0.5\right)}^{2}}{3}\right]}^{\frac{1}{3}}=0 \end{array}$$

Similarly, for an IvNS *H* with *h_i_* = <*a_i_,* [1, 1], [0, 0], [0, 0]> or *h_i_* = <*a_i_,* [0, 0], [0, 0], [1, 1]> (*i* = 1, 2, ..., *n*), by Equation (6) we have:

$$EB_{2}(H)=1-\frac{1}{n}\sum_{i=1}^{n}{\left[\frac{\begin{array}{l} 4{\left(T_{H}^{-}(a_{i})-0.5\right)}^{2}+4{\left(T_{H}^{+}(a_{i})-0.5\right)}^{2}\\ +4{\left(U_{H}^{-}(a_{i})-0.5\right)}^{2}+4{\left(U_{H}^{+}(a_{i})-0.5\right)}^{2}\\ +4{\left(F_{H}^{-}(a_{i})-0.5\right)}^{2}+4{\left(F_{H}^{+}(a_{i})-0.5\right)}^{2} \end{array}}{6}\right]}^{\frac{1}{3}}=1-\frac{1}{n}\sum_{i=1}^{n}{\left[\frac{\begin{array}{l} 4{\left(1-0.5\right)}^{2}+4{\left(1-0.5\right)}^{2}\\ +4{\left(0-0.5\right)}^{2}+4{\left(0-0.5\right)}^{2}\\ +4{\left(0-0.5\right)}^{2}+4{\left(0-0.5\right)}^{2} \end{array}}{6}\right]}^{\frac{1}{3}}=0$$

$$\mathrm{or}\;EB_{2}(H)=1-\frac{1}{n}\sum_{i=1}^{n}{\left[\frac{\begin{array}{l} 4{\left(T_{H}^{-}(a_{i})-0.5\right)}^{2}+4{\left(T_{H}^{+}(a_{i})-0.5\right)}^{2}\\ +4{\left(U_{H}^{-}(a_{i})-0.5\right)}^{2}+4{\left(U_{H}^{+}(a_{i})-0.5\right)}^{2}\\ +4{\left(F_{H}^{-}(a_{i})-0.5\right)}^{2}+4{\left(F_{H}^{+}(a_{i})-0.5\right)}^{2} \end{array}}{6}\right]}^{\frac{1}{3}}=1-\frac{1}{n}\sum_{i=1}^{n}{\left[\frac{\begin{array}{l} 4{\left(0-0.5\right)}^{2}+4{\left(0-0.5\right)}^{2}\\ +4{\left(0-0.5\right)}^{2}+4{\left(0-0.5\right)}^{2}\\ +4{\left(1-0.5\right)}^{2}+4{\left(1-0.5\right)}^{2} \end{array}}{6}\right]}^{\frac{1}{3}}=0$$

(EBP2) Let $f(x_{i})=4{\left(x_{i}-0.5\right)}^{2}$ be a function for *x_i_* ∊ [0, 1] (*i* = 1, 2, ..., *n*). It is clear that the minimum value of *f(x_i_)* = 0 can be gotten at the critical point *x_i_* = 0.5. Based on the function *f*(*x_i_*), by Equations (5) and (6) the dimension root entropy of *H* can be rewritten as the following form:

$$EB_{1}(H)=1-\frac{1}{n}\sum_{i=1}^{n}{\left[\frac{f(T_{H}(a_{i}))+f(U_{H}(a_{i}))+f(F_{H}(a_{i}))}{3}\right]}^{\frac{1}{3}}\;\mathrm{for}\;\mathrm{the}\;\mathrm{SvNS}\;H,$$

$$EB_{2}(H)=1-\frac{1}{n}\sum_{i=1}^{n}{\left[\frac{\begin{array}{l} f(T_{H}^{-}(a_{i}))+f(T_{H}^{+}(a_{i}))\\ +f(U_{H}^{-}(a_{i}))+f(U_{H}^{+}(a_{i}))\\ +f(F_{H}^{-}(a_{i}))+f(F_{H}^{+}(a_{i})) \end{array}}{6}\right]}^{\frac{1}{3}}\;\mathrm{for}\;\mathrm{the}\;\mathrm{IvNS}\;H.$$

Obviously, if and only if *T_H_*(*a_i_*) = *U_H_*(*a_i_*) = *F_H_*(*a_i_*) = 0.5, the maximum value of the entropy measure is *EB*_1_(*H*) *=* 1; while if and only if $T_{H}^{-}\left(a_{i}\right)=T_{H}^{+}\left(a_{i}\right)=U_{H}^{-}\left(a_{i}\right)=U_{H}^{+}\left(a_{i}\right)=F_{H}^{-}\left(a_{i}\right)=F_{H}^{+}\left(a_{i}\right)=0.5$, the maximum value of the entropy measure is *EB*_2_(*H*) = 1.

Thus, the property EBP2 can hold for the dimension root entropy.

(EBP3) It is obvious that $f(x_{i})=4{\left(x_{i}-0.5\right)}^{2}$ is monotonically decreasing when *x_i_* ∊ [0, 0.5], and monotonically increasing when *x_i_* ∊ [0.5, 1]. Therefore, the closer the simplified NS *H* is to the fuzziest simplified NS *B* than a simplified NS *L*, the fuzzier *H* is than *L* with *EB_k_*(*L*) < *EB_k_*(*H*) *for k =* 1, 2*.*

(EBP4) Since the complement of the SvNS *H* = {<*a_i_*, *T_H_*(*a_i_*), *U_H_*(*a_i_*), *F_H_*(*a_i_*)> | *a_i_* ∊ *A*} is *H^C^*= {<*a_i_*, *F_H_*(*a_i_*), 1 – *U_H_*(*a_i_*), *T_H_*(*a_i_*)> | *a_i_* ∊ *A*}, by Equation (5) we have:

$$\begin{array}{l} EB_{1}(H^{C})=1-\frac{1}{n}\sum_{i=1}^{n}{\left[\frac{4{\left(F_{i}-0.5\right)}^{2}+4{\left(1-U_{i}-0.5\right)}^{2}+4{\left(T_{i}-0.5\right)}^{2}}{3}\right]}^{\frac{1}{3}}\\ =1-\frac{1}{n}\sum_{i=1}^{n}{\left[\frac{4{\left(T_{i}-0.5\right)}^{2}+4{\left(U_{i}-0.5\right)}^{2}+4{\left(F_{i}-0.5\right)}^{2}}{3}\right]}^{\frac{1}{3}}=EB_{1}(H) \end{array}$$

When the complement of the IvNS $H=\left\{<a_{i},[T_{H}^{-}(a_{i}),T_{H}^{+}(a_{i})],[U_{H}^{-}(a_{i}),U_{H}^{+}(a_{i})],[F_{H}^{-}(a_{i}),F_{H}^{+}(a_{i})]>\;|a_{i}\in A\right\}$ is $H^{C}=\left\{<a_{i},[F_{H}^{-}(a_{i}),F_{H}^{+}(a_{i})],[1-U_{H}^{-}(a_{i}),1-U_{H}^{+}(a_{i})],[T_{H}^{-}(a_{i}),T_{H}^{+}(a_{i})]>\;|a_{i}\in A\right\}$, we can also obtain $EB_{2}(H^{C})=EB_{2}(H)$.

Thus, the proof of the theorem is completed.  □

## 4. Comparative Analysis of Entropy Measures for IvNSs

The comparative analysis between the presented simplified neutrosophic entropy measures and the existing entropy measures of simplified NSs are shown in this section. Since SvNS is a special case of IvNS when the two bounded values of its each interval are the same, the example adopted from [21] was illustrated only in IvNS setting. Then the existing entropy measures [19,20,21,22,23] of the IvNS *H* used for the comparison are introduced as follows:

(7) $$R_{1}(H)=1-\frac{1}{3n}\sum_{i=1}^{n}\left[\begin{array}{l} \left|T_{H}^{-}(a_{i})-0.5|+|T_{H}^{+}(a_{i})-0.5\right|\\ +|U_{H}^{-}(a_{i})-0.5|+|U_{H}^{+}(a_{i})-0.5|\\ +|F_{H}^{-}(a_{i})-0.5|+|F_{H}^{+}(a_{i})-0.5| \end{array}\right],$$

(8) $$R_{2}(H)=1-2{\left\{\frac{1}{6n}\sum_{i=1}^{n}\left[\begin{array}{l} {\left(T_{H-}(a_{i})-0.5\right)}^{2}+{\left(T_{H+}(a_{i})-0.5\right)}^{2}\\ +{\left(U_{H-}(a_{i})-0.5\right)}^{2}+{\left(U_{H+}(a_{i})-0.5\right)}^{2}\\ +{\left(F_{H-}(a_{i})-0.5\right)}^{2}+{\left(F_{H+}(a_{i})-0.5\right)}^{2} \end{array}\right]\right\}}^{1/2},$$

(9) $$R_{3}(H)=1-\frac{2}{3n}\sum_{i=1}^{n}\left\{\begin{array}{l} \max\left[\left|T_{H}^{-}(a_{i})-0.5|,|T_{H}^{+}(a_{i})-0.5\right|\right]+\\ \max\left[\left|U_{H}^{-}(a_{i})-0.5|,|U_{H}^{+}(a_{i})-0.5\right|\right]\\ +\max\left[\left|F_{H}^{-}(a_{i})-0.5|,|F_{H}^{+}(a_{i})-0.5\right|\right] \end{array}\right\},$$

(10) $$R_{4}(H)=1-\frac{2}{n}\sum_{i=1}^{n}\left\{\max\left[\begin{array}{l} \frac{1}{2}(|T_{H}^{-}(a_{i})-0.5|+|T_{H}^{+}(a_{i})-0.5|),\\ \frac{1}{2}(|U_{H}^{-}(a_{i})-0.5|+|U_{H}^{+}(a_{i})-0.5|),\\ \frac{1}{2}(|F_{H}^{-}(a_{i})-0.5|+|F_{H}^{+}(a_{i})-0.5|) \end{array}\right]\right\},$$

(11) $$R_{5}(H)=1-\frac{1}{2n}\sum_{i=1}^{n}\left\{\begin{array}{l} \left[T_{H}^{-}(a_{i})+F_{H}^{-}(a_{i})]|U_{H}^{-}(a_{i})-{\left(U_{H}^{-}(a_{i})\right)}^{c}\right|\\ +[T_{H}^{+}(a_{i})+F_{H}^{+}(a_{i})]|U_{H}^{+}(a_{i})-{\left(U_{H}^{+}(a_{i})\right)}^{c}| \end{array}\right\},$$

(12) $$R_{6}(H)=\frac{1}{n}\sum_{i=1}^{n}\frac{2-|T_{H}^{-}(a_{i})-F_{H}^{-}(a_{i})|-|T_{H}^{+}(a_{i})-F_{H}^{+}(a_{i})|-|U_{H}^{-}(a_{i})-U_{H}^{+}(a_{i})|}{2+|T_{H}^{-}(a_{i})-F_{H}^{-}(a_{i})|+|T_{H}^{+}(a_{i})-F_{H}^{+}(a_{i})|+|U_{H}^{-}(a_{i})-U_{H}^{+}(a_{i})|},$$

(13) $$R_{7}(H)=\frac{1}{6n(\sqrt{e}-1)}\sum_{i=1}^{n}[\begin{array}{c} T_{H}^{-}(a_{i})e^{\left(1-T_{H}^{-}(a_{i})\right)}+(1-T_{H}^{-}(a_{i}))e^{T_{H}^{-}(a_{i})}-1\\ +U_{H}^{-}(a_{i})e^{\left(1-U_{H}^{-}(a_{i})\right)}+(1-U_{H}^{-}(a_{i}))e^{U_{H}^{-}(a_{i})}-1\\ +F_{H}^{-}(a_{i})e^{\left(1-F_{H}^{-}(a_{i})\right)}+(1-F_{H}^{-}(a_{i}))e^{F_{H}^{-}(a_{i})}-1\\ +T_{H}^{+}(a_{i})e^{\left(1-T_{H}^{+}(a_{i})\right)}+(1-T_{H}^{+}(a_{i}))e^{T_{H}^{+}(a_{i})}-1\\ +U_{H}^{+}(a_{i})e^{\left(1-U_{H}^{+}(a_{i})\right)}+(1-U_{H}^{+}(a_{i}))e^{U_{H}^{+}(a_{i})}-1\\ +F_{H}^{+}(a_{i})e^{\left(1-F_{H}^{+}(a_{i})\right)}+(1-F_{H}^{+}(a_{i}))e^{F_{H}^{+}(a_{i})}-1 \end{array}]$$

(14) $$R_{8}(H)=\frac{1}{6n}\sum_{i=1}^{n}\left[\begin{array}{l} \sin(T_{H}^{-}(a_{i})\pi )+\sin(T_{H}^{+}(a_{i})\pi )\\ +\sin(U_{H}^{-}(a_{i})\pi )+\sin(U_{H}^{+}(a_{i})\pi )\\ +\sin(F_{H}^{-}(a_{i})\pi )+\sin(F_{H}^{-}(a_{i})\pi ) \end{array}\right].$$

Assume an IvNS is $H=\left\{<a_{i},[T_{H}^{-}(a_{i}),T_{H}^{+}(a_{i})],[U_{H}^{-}(a_{i}),U_{H}^{+}(a_{i})],[F_{H}^{-}(a_{i}),F_{H}^{+}(a_{i})]>\;|a_{i}\in A\right\}$ in the universal set *A* = {*a*_1_, *a*_2_, ..., *a_n_*}. Then, *H^n^* for *n >* 0 can be expressed as:

(15) $$H^{n}=\{<a_{i},[{\left(T_{H}^{-}(a_{i})\right)}^{n},{\left(T_{H}^{+}(a_{i})\right)}^{n}],[1-{\left(1-U_{H}^{-}(a_{i})\right)}^{n},1-{\left(1-U_{H}^{+}(a_{i})\right)}^{n}],[1-{\left(1-F_{H}^{-}(a_{i})\right)}^{n},1-{\left(1-F_{H}^{+}(a_{i})\right)}^{n}]>\;|a_{i}\in A\}.$$

Provided that an IvNS *H* in *A* = {*a*_1_, *a*_2_, *a*_3_, *a*_4_, *a*_5_} = {1, 2, 3, 4, 5} is evaluated by *H* = {<1, [0.2, 0.3], [0.6, 0.6], [0.7, 0.8]>, <2, [0.3, 0.3], [0.5, 0.6], [0.5, 0.6]>, <3, [0.4, 0.5], [0.5, 0.5], [0, 0.1]>, <4, [1, 1], [0.4, 0.4], [0, 0.1]>, <5, [0.7, 0.8], [0.5, 0.5], [0, 0]>}, then regarding the characteristics of variables corresponding to these operations [21]: (1) *H* can be regarded as “large” in *A*; (2) *H*^2^ can be regarded as “very large”; (3) *H*^3^ can be regarded as “quite very large”; (4) *H*^4^ can be regarded as “very very large”. Then the operational results are shown in Table 1.

**Figure 1:**
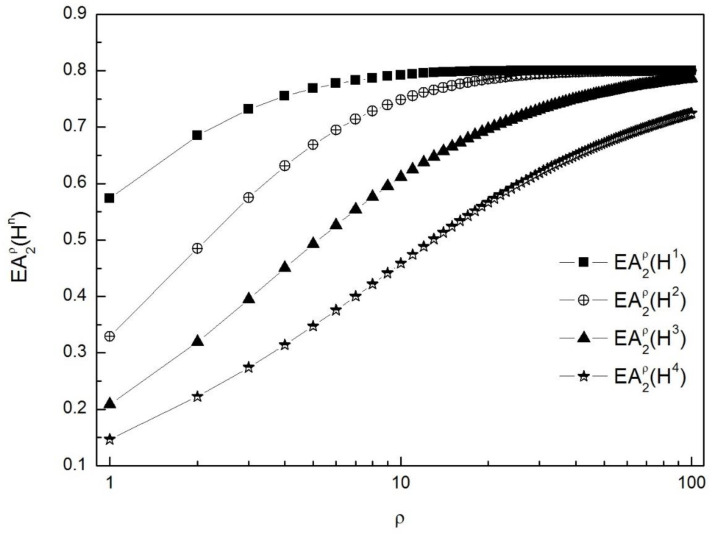
The entropy measure curves of $EA_{2}^{\rho }(H^{n})$ for *H*^1^*, H*^2^*, H*^3^, *H*^4^ and *ρ* ∊ [1, 100].

Then, calculated by Equations (2) and (6)–(14), the measure values of the relative entropy are shown in Table 2. Then, the entropy measure curves of $EA_{2}^{\rho }(H^{n})$ for *n* = 1, 2, 3, 4 and *ρ* ∊ [1, 100] are also shown in Figure 1.

Then, the available entropy measures of IvNSs should satisfy the ranking order $EA_{2}^{\rho }(H)$ > $EA_{2}^{\rho }(H^{2})$ > $EA_{2}^{\rho }(H^{3})$ > $EA_{2}^{\rho }(H^{4})$ from the intuition*.* From Table 2, except for both $EA_{2}^{100}(H^{n})$ with the ranking order $EA_{2}^{100}(H)$ = $EA_{2}^{100}(H^{2})$ > $EA_{2}^{100}(H^{3})$ > $EA_{2}^{100}(H^{4})$ and *R*_6_(*H^n^*) with the ranking order *R*_6_(*H*) > *R*_6_(*H*^4^) > *R*_6_(*H*^3^) > *R*_6_(*H*^2^)*,* the entropy measure values of $EA_{2}^{\rho }(H^{n})$ for *ρ* ∊ [1, 100) and *EB*_2_(*H^n^*) and the existing *R*_1_(*H^n^*)*–R*_5_(*H^n^*)*, R*_7_(*H^n^*)*, R*_8_(*H^n^*) satisfy the above ranking demand of the available entropy measures. Furthermore, from Figure 1, the ranking order based on the entropy measure values of $EA_{2}^{\rho }(H^{n})$ indicates some robustness regarding *ρ* from 1 to 100. However, with the parameter *ρ* increasing, especially when *ρ >* 30*,* the entropy measure values of $EA_{2}^{\rho }(H)$ and $EA_{2}^{\rho }(H^{2})$ tend to the same value 0.8.

## 5. Decision-Making Example Using Simplified Neutrosophic Entropy in IvNS Setting

In this section, the proposed entropy measures are applied in a decision-making problem, and then compared with the existing entropy measures. For convenience, an investment decision-making example adopted from the reference [21] was used for the application. In the decision-making problem, the decision makers are requested to assess four investment projects (alternatives), including a clothing company (*g*_1_), a food company (*g*_2_), a computer company (*g*_3_), and a house-building company (*g*_4_), over three attributes, like growth (*a*_1_), risk (*a*_2_), and environmental impact (*a*_3_) respectively, and then select the best alternative for the investment company. The evaluation information of the alternative set *G* = {*g*_1_, *g*_2_, *g*_3_, *g*_4_} over the attribute set *A* = {*a*_1_, *a*_2_, *a*_3_} is given by the form of IvNSs as the following matrix:

$$M=\begin{array}{c} g_{1}\\ g_{2}\\ g_{3}\\ g_{4} \end{array}[\begin{array}{c} <a_{1},[0.4,0.6],[0.1,0.3],[0.2,0.3]>\\ <a_{1},[0.3,0.6],[0.3,0.5],[0.8,0.9]>\\ <a_{1},[0.5,0.6],[0.2,0.3],[0.3,0.4]>\\ <a_{1},[0.5,0.6],[0.3,0.4],[0.8,0.9]> \end{array}\begin{array}{c} <a_{2},[0.7,0.9],[0.2,0.3],[0.4,0.5]>\\ <a_{2},[0.6,0.7],[0.1,0.2],[0.2,0.3]>\\ <a_{2},[0.3,0.6],[0.2,0.3],[0.3,0.4]>\\ <a_{2},[0.7,0.8],[0,0.1],[0.1,0.2]> \end{array}\begin{array}{c} <a_{3},[0.4,0.5],[0.2,0.3],[0.3,0.4]>\\ <a_{3},[0.6,0.7],[0.1,0.2],[0.2,0.3]>\\ <a_{3},[0.4,0.5],[0.2,0.5],[0.7,0.9]>\\ <a_{3},[0.6,0.7],[0.1,0.2],[0.1,0.3]> \end{array}]$$

By applying the proposed entropy measures of Equations (2) and (6) and the existing entropy measures of Equations (7)–(14) to the above decision-making problem, the relative entropy measure results and the ranking orders are listed in Table 3.

Obviously, the entropy measure values of $EA_{2}^{\rho }(g_{i})$ (*i* =1, 2, 3, 4) for *ρ* ≤ 20 indicate the identical ranking result *g*_3_ > *g*_1_ > *g*_2_ > *g*_4_, and then it is changed into *g*_3_ = *g*_1_ > *g*_2_ > *g*_4_ for *ρ* = 30 and gets *g*_3_ = *g*_1_ = *g*_2_ > *g*_4_ along with the parameter values of *ρ* ranging from 40 to 100. Obviously, $EA_{2}^{\rho }(g_{i})$ is a better selection with relatively small values of *ρ* such as *ρ* ≤ 20. On the other hand, the measure values of the proposed entropy *EB*_2_(*g_i_*) (*i* = 1, 2, 3, 4) and existing entropy including *R*_1_(*g_i_*)*-R*_5_(*g_i_*), *R*_7_(*g_i_*) have the same ranking order *g*_3_ > *g*_1_ > *g*_2_ > *g*_4_; while that of *R*_6_(*g_i_*) is *g*_3_ > *g*_1_ > *g*_4_ > *g*_2_*.* Anyway, the best alternative of all the ranking orders is *g*_3_.

## 6. Conclusions

This study originally presented the generalized distance-based entropy measure and the dimension root entropy measure of simplified NSs, containing both the SvSN and IvSN generalized distance-based entropy measures and the SvSN and IvSN neutrosophic dimension root entropy measures. Then, their properties were discussed based on the axioms of an entropy measure of IvNSs defined in [21]. After that, a comparison between the proposed entropy and existing relative entropy measures by a numerical example in IvNS setting showed that the proposed entropy measures are effective and rational. An application of the proposed two entropy measures in an actual decision-making problem illustrated the feasibility and rationality by comparison with the existing ones, especially with the relatively small values of the parameter *ρ*, such as *ρ* < 20. The proposed simplified NS entropy not only is a complement of the entropy theory of simplified NSs, but also presents a new effective way of the uncertain measure under the simplified NS setting. Our future work will focus on research to extend the proposed entropy measures to applications in diverse engineering fields.

## Figures and Tables

**Table 1:** Operation results of *H^n^* for *n* = 1, 2, 3, 4.

*H^n^*	*a*_1_ = 1	*a*_2_ = 2	*a*_3_ = 3	*a*_4_ = 4	*a*_5_ = 5
*H*	<1,[0.2, 0.3], [0.6, 0.6], [0.7, 0.8]>	<2,[0.3, 0.3], [0.5, 0.6], [0.5, 0.6]>	<3,[0.4, 0.5], [0.5, 0.5], [0, 0.1]>	<4,[1, 1], [0.4, 0.4], [0, 0.1]>	<5,[0.7, 0.8], [0.5, 0.5], [0, 0]>
*H* ^2^	<1,[0.04, 0.09], [0.84, 0.84], [0.91, 0.96]>	<2,[0.09, 0.09], [0.75, 0.84], [0.75, 0.84]>	<3,[0.16, 0.25], [0.75, 0.75], [0, 0.19]>	<4,[1, 1], [0.64, 0.64], [0, 0.19]>	<5,[0.49, 0.64], [0.75, 0.75], [0, 0]>
*H* ^3^	<1,[0.008, 0.027], [0.936, 0.936], [0.973, 0.992]	<2,[0.027, 0.027], [0.875, 0.936], [0.875, 0.936]>	<3,[0.064, 0.125], [0.875, 0.875], [0, 0.271]>	<4,[1, 1], [0.784, 0.784], [0, 0.271]>	<5,[0.343, 0.512], [0.875, 0.875], [0, 0]>
*H* ^4^	<1,[0.0016, 0.0081], [0.9744, 0.9744], [0.9919, 0.9984]>	<2,[0.0081, 0.0081], [0.9375, 0.9744], [0.9375, 0.9744]>	<3,[0.0256, 0.0625], [0.9375, 0.9375], [0, 0.3439]>	<4,[1, 1], [0.8704, 0.8704], [0, 0.3439]>	<5,[0.2401, 0.4096], [0.9375, 0.9375], [0, 0]>

**Table 2:** All values of various entropy measures of IvNSs.

Entropy Value	*H*	*H* ^2^	*H* ^3^	*H* ^4^	Ranking Order
$EA_{2}^{1}(H^{n})$	0.5733	0.3293	0.2083	0.1465	$EA_{2}^{1}(H)$ > $EA_{2}^{1}(H^{2})$ > $EA_{2}^{1}(H^{3})$ > $EA_{2}^{1}(H^{4})$
$EA_{2}^{2}(H^{n})$	0.6853	0.485	0.3193	0.2228	$EA_{2}^{2}(H)$ > $EA_{2}^{2}(H^{2})$ > $EA_{2}^{2}(H^{3})$ > $EA_{2}^{2}(H^{4})$
$EA_{2}^{3}(H^{n})$	0.7317	0.5749	0.395	0.2743	$EA_{2}^{3}(H)$ > $EA_{2}^{3}(H^{2})$ > $EA_{2}^{3}(H^{3})$ > $EA_{2}^{3}(H^{4})$
$EA_{2}^{4}(H^{n})$	0.7551	0.6313	0.4503	0.3143	$EA_{2}^{4}(H)$ > $EA_{2}^{4}(H^{2})$ > $EA_{2}^{4}(H^{3})$ > $EA_{2}^{4}(H^{4})$
$EA_{2}^{5}(H^{n})$	0.7686	0.6688	0.4927	0.3473	$EA_{2}^{5}(H)$ > $EA_{2}^{5}(H^{2})$ > $EA_{2}^{5}(H^{3})$ > $EA_{2}^{5}(H^{4})$
$EA_{2}^{10}(H^{n})$	0.7922	0.7484	0.611	0.4589	$EA_{2}^{10}(H)$ > $EA_{2}^{10}(H^{2})$ > $EA_{2}^{10}(H^{3})$ > $EA_{2}^{10}(H^{4})$
$EA_{2}^{20}(H^{n})$	0.7992	0.7848	0.6963	0.5667	$EA_{2}^{20}(H)$ > $EA_{2}^{20}(H^{2})$ > $EA_{2}^{20}(H^{3})$ > $EA_{2}^{20}(H^{4})$
$EA_{2}^{30}(H^{n})$	0.7999	0.7942	0.7309	0.6191	$EA_{2}^{30}(H)$ > $EA_{2}^{30}(H^{2})$ > $EA_{2}^{30}(H^{3})$ > $EA_{2}^{30}(H^{4})$
$EA_{2}^{40}(H^{n})$	0.8	0.7976	0.7499	0.6505	$EA_{2}^{40}(H)$ > $EA_{2}^{40}(H^{2})$ > $EA_{2}^{40}(H^{3})$ > $EA_{2}^{40}(H^{4})$
$EA_{2}^{50}(H^{n})$	0.8	0.799	0.7618	0.6719	$EA_{2}^{50}(H)$ > $EA_{2}^{50}(H^{2})$ > $EA_{2}^{50}(H^{3})$ > $EA_{2}^{50}(H^{4})$
$EA_{2}^{100}(H^{n})$	0.8	0.8	0.7862	0.7247	$EA_{2}^{100}(H)$ = $EA_{2}^{100}(H^{2})$ > $EA_{2}^{100}(H^{3})$ > $EA_{2}^{100}(H^{4})$
*EB*_2_(*H^n^*)	0.3534	0.2013	0.1231	0.0829	*EB*_2_(*H*) > *EB*_2_(*H*^2^) > *EB*_2_(*H*^3^) > *EB*_2_(*H*^4^)
*R*_1_(*H^n^*) [21]	0.5733	0.3293	0.2083	0.1465	*R*_1_(*H*) > *R*_1_(*H*^2^) > *R*_1_(*H*^3^) > *R*_1_(*H*^4^)
*R*_2_(*H^n^*) [21]	0.439	0.2824	0.175	0.1184	*R*_2_(*H*) > *R*_2_(*H*^2^) > *R*_2_(*H*^3^) > *R*_2_(*H*^4^)
*R*_3_(*H^n^*) [21]	0.52	0.2707	0.1477	0.0811	*R*_3_(*H*) > *R*_3_(*H*^2^) > *R*_3_(*H*^3^) > *R*_3_(*H*^4^)
*R*_4_(*H^n^*) [21]	0.24	0.1	0.0556	0.0228	*R*_4_(*H*) > R_4_(*H*^2^) > R_4_(*H*^3^) > R_4_(*H*^4^)
*R*_5_(*H^n^*) [19,21]	0.9	0.6109	0.4464	0.366	*R*_5_(*H*) > *R*_5_(*H*^2^) > *R*_5_(*H*^3^) > *R*_5_(*H*^4^)
*R*_6_(*H^n^*) [20]	0.2938	0.2684	0.2698	0.2719	*R*_6_(*H*) > *R*_6_(*H*^4^) > *R*_6_(*H*^3^) > *R*_6_(*H*^2^)
*R*_7_(*H^n^*) [22]	0.6886	0.4919	0.3255	0.2272	*R*_7_(*H*) > *R*_7_(H^2^) > *R*_7_(*H*^3^) > *R*_7_(*H*^4^)
*R*_8_(*H^n^*) [23]	0.6695	0.4521	0.2902	0.2027	*R*_8_(*H*) > *R*_8_(*H*^2^) > *R*_8_(*H*^3^) > *R*_8_(*H*^4^)

**Table 3:** All the results of the proposed entropy and existing entropy measures of IvNSs.

$EA_{2}^{\rho }(g_{i})$	*g*_1_	*g*_2_	*g*_3_	*g*_4_	Ranking Order
$EA_{2}^{1}(g_{i})$	0.6333	0.5333	0.6556	0.4444	*g*_3_ > *g*_1_ > *g*_2_ > *g*_4_
$EA_{2}^{2}(g_{i})$	0.8111	0.7333	0.8378	0.6356	*g*_3_ > *g*_1_ > *g*_2_ > *g*_4_
$EA_{2}^{3}(g_{i})$	0.8867	0.832	0.9116	0.7351	*g*_3_ > *g*_1_ > *g*_2_ > *g*_4_
$EA_{2}^{4}(g_{i})$	0.9252	0.8869	0.9466	0.7945	*g*_3_ > *g*_1_ > *g*_2_ > *g*_4_
$EA_{2}^{5}(g_{i})$	0.9477	0.9203	0.9653	0.8332	*g*_3_ > *g*_1_ > *g*_2_ > *g*_4_
$EA_{2}^{10}(g_{i})$	0.987	0.9804	0.993	0.9132	*g*_3_ > *g*_1_ > *g*_2_ > *g*_4_
$EA_{2}^{20}(g_{i})$	0.9987	0.9981	0.9994	0.9412	*g*_3_ > *g*_1_ > *g*_2_ > *g*_4_
$EA_{2}^{30}(g_{i})$	0.9999	0.9998	0.9999	0.9441	*g*_3_ = *g*_1_ > *g*_2_ > *g*_4_
$EA_{2}^{40}(g_{i})$	1	1	1	0.9444	*g*_3_ = *g*_1_ = *g*_2_ > *g*_4_
$EA_{2}^{50}(g_{i})$	1	1	1	0.9444	*g*_3_ = *g*_1_ = *g*_2_ > *g*_4_
$EA_{2}^{100}(g_{i})$	1	1	1	0.9444	*g*_3_ = *g*_1_ = *g*_2_ > *g*_4_
*EB*_2_(*g_i_*)	0.4302	0.3572	0.4571	0.2945	*g*_3_ > *g*_1_ > *g*_2_ > *g*_4_
*R*_1_(*g_i_*) [21]	0.6333	0.5333	0.6556	0.4444	*g*_3_ > *g*_1_ > *g*_2_ > *g*_4_
*R*_2_(*g_i_*) [21]	0.5654	0.4836	0.5972	0.3963	*g*_3_ > *g*_1_ > *g*_2_ > *g*_4_
*R*_3_(*g_i_*) [21]	0.5111	0.4222	0.5333	0.3333	*g*_3_ > *g*_1_ > *g*_2_ > *g*_4_
*R*_4_(*g_i_*) [21]	0.4333	0.3	0.4667	0.2333	*g*_3_ > *g*_1_ > *g*_2_ > *g*_4_
*R*_5_(*g_i_*) [19,21]	0.52	0.5133	0.57	0.3933	*g*_3_ > *g*_1_ > *g*_2_ > *g*_4_
*R*_6_(*g_i_*) [20]	0.5687	0.364	0.5728	0.3818	*g*_3_ > *g*_1_ > *g*_4_ > *g*_2_
*R*_7_(*g_i_*) [22]	0.8165	0.7406	0.8429	0.6431	*g*_3_ > *g*_1_ > *g*_2_ > *g*_4_
*R*_8_(*g_i_*) [23]	0.7852	0.6985	0.8129	0.5997	*g*_3_ > *g*_1_ > *g*_2_ > *g*_4_
